# Untargeted LC-HRMS-Based Metabolomic and Antibacterial Potential of *Sargassum duplicatum* Against Multidrug-Resistant Bacteria

**DOI:** 10.3390/medicina62010218

**Published:** 2026-01-20

**Authors:** Feri Susanto, Riyanti, Hamdan Syakuri, Muhammad Nursid, Till F. Schäberle, Ute Mettal, Jae-Suk Choi, Maria Dyah Nur Meinita

**Affiliations:** 1 Faculty of Fisheries and Marine Science, Jenderal Soedirman University, Purwokerto 53123, Indonesia; 2 Agricultural Biotechnology Magister Program, Graduate School, Jenderal Soedirman University, Purwokerto 53123, Indonesia; 3 Research Center for Food Technology and Processing, National Research and Innovation Agency (BRIN), Playen, Yogyakarta 55861, Indonesia; 4 Faculty of Agricultural Sciences, Nutritional Sciences and Environmental Management, Justus Liebig University, 35390 Giessen, Germany; 5 Natural Product Department, Fraunhofer-Institute for Molecular Biology and Applied Ecology (IME), Ohlebergsweg 12, 35392 Giessen, Germany; 6 Department of Seafood Science and Technology, The Institute of Marine Industry, Gyeongsang National University, 38 Cheondaegukchi-gil, Tongyeong-si 53064, Gyeongsangnam-do, Republic of Korea

**Keywords:** seaweed, molecular, metabolomic, antimicrobial, algae, MDR, GNPS, LC-HRMS

## Abstract

***Background/Objectives:*** The rise in antimicrobial resistance is one of the major challenges to global health systems, which necessitates the development of new antibacterial compounds. The bioactive compounds of brown seaweed *Sargassum duplicatum* have demonstrated potential antibacterial activity. This study applied metabolomic profiling and molecular networking in combination with antibacterial screening assays to assess the antimicrobial properties of *S. duplicatum* extracts against multidrug-resistant bacteria. ***Methods:*** Two extraction methods, i.e., maceration and microwave extraction, were used. Therewith, untargeted metabolomic profiling was performed using Liquid Chromatography–High Resolution Mass Spectrometry (LC-HRMS). Molecular networks (MNs) were established and compound dereplication was conducted using the spectral database of the Global Natural Products Social Molecular Networking platform (GNPS). Additionally, antimicrobial assays were conducted against Gram-positive and Gram-negative bacterial strains, including multidrug-resistant bacteria, i.e., methicillin-resistant *Staphyloccocus aureus* ATCC 33592 (MRSA) and β-lactamase, producing *Escherichia coli* ATCC 35218 (TEM-1 positive strain). ***Result:*** Dereplication resulted in the prediction of six compounds with reported antimicrobial properties, i.e., 13-docosenamide, 9-octadecenamide, pheophorbide A, ouabain, sarmentoside B and AC1L1X1Z. Antibacterial screening of the extracts revealed that the ethyl acetate maceration extracts exhibited the strongest inhibitory activity, with inhibition values between 85 and 98% against *S. aureus* ATCC 33592. ***Conclusions:*** This metabolomics study requires further research to isolate, purify, confirm, and validate the dereplicated compounds that may have potential antibacterial activity.

## 1. Introduction

Antimicrobial resistance (AMR) is a global health threat that calls for immediate action. Previous studies have projected the impact of AMR on human health and mortality [1,2,3]. According to the most recent projections, by 2050, antimicrobial resistance could directly result in 1.91 million deaths each year, with a further 8.22 million deaths indirectly linked to AMR [4]. The failure to implement effective countermeasures against AMR will result in mortality rates exceeding those of cancer by 2050, alongside cumulative economic costs surpassing USD 100 trillion globally [5,6].

Traditional antibiotics are losing their effectiveness as microbes develop resistance to the antibiotics currently widely available on the commercial market. The primary solution underway in AMR research is the discovery and development of new antimicrobial drugs from potential natural resources. Discovering and identifying new antimicrobial compounds is crucial due to the rapid increase in AMR and the dwindling availability of effective antibiotics. However, traditional exploration and screening of antimicrobial compounds from terrestrial resources often leads to the “rediscovery” of already known compounds, leading to redundancy [7,8].

The high diversity of marine sources offers promising opportunities for the discovery of novel bioactive compounds that might have the potential to contribute to the discovery of lead structures with the goal to develop compounds that can contribute to combat antimicrobial-resistant pathogens. Brown seaweed (Phaeophyta), an abundant marine resource, holds a putative potential as a source of bioactive compounds. Phaeophyta has the ability to connect oxidative reactions caused by sunlight and air with the halogenation of different substances [9]. Among the Phaeophyta species, *S. duplicatum* has gained considerable attention due to its diverse bioactive metabolites, which show promising antimicrobial properties [10,11,12,13]. Exploring the vast diversity of bioactive compounds from marine macroalgae requires novel techniques that can be performed faster and in higher throughput than classical approaches [14]. Metabolomic analysis can be performed a in high-throughput capacity, allows for the exploration of a large number of metabolites, even if they are produced in low concentrations, and can be performed in a shorter time [14,15].

The advancement of untargeted Liquid Chromatography–High Resolution Mass Spectrometry (LC-HRMS)-based metabolomic profiling has enhanced the integration of sophisticated diagnostic tools within clinical laboratories [15]. This technique not only offers high analytical specificity and the capability to analyze multiple metabolites concurrently. Furthermore, due to the precise mass measurement, it facilitates untargeted identification of compounds [16]. This study aimed to observe untargeted putative compounds present in *S. duplicatum* that could potentially demonstrate antimicrobial properties. Two different methods were used for extract generation, i.e., maceration and microwave extraction (ME). To analyze the antibacterial properties of the extracts, screening was performed against methicillin-resistant *S. aureus* (MRSA) and drug-resistant *E. coli* strains, which are among leading pathogens for deaths associated with multidrug-resistant bacteria [3,4]. MRSA resistance occurs through the acquisition of the mecA gene, which encodes an altered penicillin-binding protein (PBP2a) with low affinity for β-lactam antibiotics such as methicillin and oxacillin [17]. While *E. coli* resistance occurs because these strains produce extended-spectrum β-lactamases (ESBLs), mechanisms such as efflux pumps, loss or mutation of porins (reducing drug entry), and biofilm formation together contribute to the ability to resist various antibiotic classes [18]. The novelty of this research is the use of an untargeted metabolomic profiling method based on LC-HRMS and the utilization of *S. duplicatum*, a marine resource that remains underexplored. These two innovations may address redundancy in antimicrobial compound discovery and optimize candidate compounds that can be developed as drugs to address AMR in the future.

## 2. Research Materials and Methods

### 2.1. Seaweed Material

Samples of *S. duplicatum* were collected during the lowest tide on shallow subtidal areas of Kukup Beach (−8.133882, 110.554731), Indonesia. The selection of sampling locations was made with consideration of the habitat representation of the *S. duplicatum* species and the environmental conditions. The collection of seaweed was conducted using non-destructive methods, ensuring that the seaweed remains undamaged and that the samples accurately represent the targeted species [19]. The seaweed samples were rinsed with sterile seawater to remove debris, epiphytes, and contaminants. Then, the seaweed were cleaned and rinsed with freshwater to eliminate salt content, debris, and epiphytes before being air-dried for 48 h and ground into powder. For metabolomic and antimicrobial studies, 2 g of dried powder sample were taken from all parts of thallus.

### 2.2. The Extraction of Bioactive Compounds

The extraction of bioactive compounds from *S. duplicatum* samples was conducted using two distinct methods: multistage maceration and the microwave extraction (ME) method. The multistage maceration technique involved the sequential extraction of *S. duplicatum* using different solvents, such as n-Hexane, ethyl acetate, methanol, and ethanol at a ratio of 1:50 for 48 h to extract non-polar, semi polar and polar compounds [20,21]. The sample was weighed, the solvent was added, and extraction was performed using a shaker for 48 h. Following filtration and evaporation, the dry extract was stored at −20 °C for subsequent analysis.

The microwave extraction method was performed to accelerate solvent penetration and enhance metabolite recovery through dielectric heating. Two grams of dried powder of *S. duplicatum* were mixed with 100 mL of solvent (n-hexane, ethyl acetate, methanol, or ethanol) and extracted using microwave irradiation at 2450 MHz and 600 W for 5 min using a microwave (Samsung, Seoul, Republic of Korea). The mixture was allowed to cool to room temperature and subsequently filtered through No. 41 filter paper (Whatman^TM^, Hangzhou, China). The filtrate was concentrated using a rotary evaporator (IKA, Staufen im Breisgau, Germany) under reduced pressure to remove the solvent, and the resulting extract was stored at −20 °C until analysis.

### 2.3. Liquid Chromatography-High Resolution Mass Spectrometry (LC-HRMS) Analysis

The identification of bioactive compounds from the extract of *S. duplicatum* was conducted by UPLC-QTOF-UHR-MS and MS/MS measurements. An extract concentration of 1 mg/mL was injected in a volume of 2 µL using methanol as the solvent [22]. A quadrupole time-of-flight spectrometer (LC-QTOF maXis II, Bruker Daltonics, Bremen, Germany) equipped with an electrospray ionization source in line with an Agilent 1290 infinity LC system (Agilent Technologies, Santa Clara, CA, USA) was used. C18 RP-UPLC (ACQUITY UPLC BEH C18 column (130 Å, 1.7 µm, 2.1 × 100 mm)) was performed at 45 °C with the following linear gradient (A: H_2_O, 0.1% HCOOH; B: CH_3_CN, 0.1% HCOOH; flow rate: 0.6 mL/min): 0 min: 95% A; 0.30 min: 95% A; 18.00 min: 4.75% A; 18.10 min: 0% A; 22.50 min: 0% A; 22.60 min: 95% A; 25.00 min: 95% A. Analytes were detected by UV absorption recorded in the range of 205−640 nm. MS spectra were recorded in positive mode using the ESI source (capillary: 4500 V, end plate offset: 500 V) over a mass range of *m*/*z* 50–2000 with a spectra rate of 1 Hz using 1.6 bar N2 nebulizer gas and 7.5 L/min N2 heated dry gas (T = 250 °C). During MS/MS experiments, spectra were recorded at a 6 Hz scan rate using collision induced fragmentation (6.0 eV collision energy, N_2_ at 10^−2^ mbar).

### 2.4. Molecular Networking Analysis

The MS/MS data obtained was converted into mzXML format from a MassHunter (.d) file using MS Convert [23]. These mzXML files are then uploaded to the Global Natural Product Social Molecular Networking online platform (https://gnps.ucsd.edu/ProteoSAFe/, accessed on 4 September 2025) based on the online workflow (https://ccms-ucsd.github.io/GNPSDocumentation/, accessed on 4 September 2025) [24]. The data was filtered by removing all MS/MS fragment ions within +/− 17 Da of the precursor *m*/*z*. MS/MS spectra were window filtered by choosing only the top 6 fragment ions in the +/− 50 Da window throughout the spectrum. The precursor ion mass tolerance was set to 2.0 Da and a MS/MS fragment ion tolerance of 0.5 Da. A network was then created where edges were filtered to have a cosine score above 0.7 and more than 6 matched peaks. Further, edges between two nodes were kept in the network if and only if each of the nodes appeared in the other’s respective top 10 most similar nodes. Finally, the maximum size of a molecular family was set to 100, and the lowest-scoring edges were removed from molecular families until the molecular family size was below this threshold. The spectra in the network were then searched against GNPS spectral libraries. The library spectra were filtered in the same manner as the input data. All matches kept between network spectra and library spectra were required to have a score above 0.7 and at least 6 matched peaks. The resulting molecular network was visualized using Cytoscape 3.10.3 software [25].

### 2.5. Antimicrobial Bioassays

The antimicrobial efficacy of crude extracts from *S. duplicatum* was evaluated using micro broth dilution assays conducted in 384-well microtiter plates (Greiner, Kremsmünster, Austria). A Cybi Liquid handling system (Analytic Jena, Jena, Germany) facilitated the distribution of 1.0, 0.5, and 0.25 μL (in duplicate, corresponding to extract concentrations of 20.0, 10.0, and 5.0 μg/mL) of each extract into the assay plates. A dilution series of gentamicin (ranging from 64 to 0.002 μg/mL, Sigma Aldrich, St. Louis, MO, USA) served as a positive control in the antibacterial assays, while wells containing only the medium or solely the bacterial suspension were designated as sterile and growth controls, respectively. Pre-cultures of *S. aureus* ATCC 33592 (MRSA) and *E. coli* ATCC35218 (TEM-1 positive strain) were incubated overnight at 37 °C with shaking at 180 rpm in cation-adjusted Mueller Hinton II medium (Becton Dickinson, Sparks, NV, USA). Following this, the cell density was adjusted to 2 × 10^4^ cells/mL, and 50 μL of the bacterial suspension was introduced into each well (excluding the sterile control) using a multi-well dispenser (Multidrop; Thermo Labsystems, Waltham, MA, USA). After an incubation period of 18 h at 37 °C with shaking at 180 rpm and 80% relative humidity, cell growth was evaluated by measuring turbidity at 600 nm using a microplate spectrophotometer (LUMIstar^®^ Omega BMG Labtech, Ortenberg, Germany) [22]. The percentage of growth inhibition was calculated using the following formula:

$$\mathrm{Growth}\;\mathrm{inhibition}\;\left(\%\right)\;=\;100\;\times \;(1–\frac{{\mathrm{OD}}_{600\;\mathrm{of sample}}-{\mathrm{OD}}_{600\;\mathrm{of only medium}}}{{\mathrm{OD}}_{600\;\mathrm{of of native control}}-{\mathrm{OD}}_{600\;\mathrm{of only medium}}})$$

### 2.6. Data Analysis

Data MassHunter (.d) files were processed with Compass DataAnalysis 4.0 to extract information regarding intensity and retention time. The intensities obtained using Compass Data Analysis 4.0 (Bruker Daltonics, Bremen, Germany) were re-visualized using RStudio 9.5 and R 4.5.1 through the Ward.D2 clustering method and Euclidean distance clustering with min-max normalization to examine the abundance of putative compounds present in each *S. duplicatum* extract. Meanwhile, statistical analysis of bacterial growth inhibition percentage was performed using the non-parametric Kruskal–Wallis test [26]. The test was conducted using SPSS 29.0 software (IBM Corp., Armonk, NY, USA). The test results were interpreted descriptively.

## 3. Results

### 3.1. LC-HRMS Metabolite Profiling and Molecular Networking

**Figure 1:**
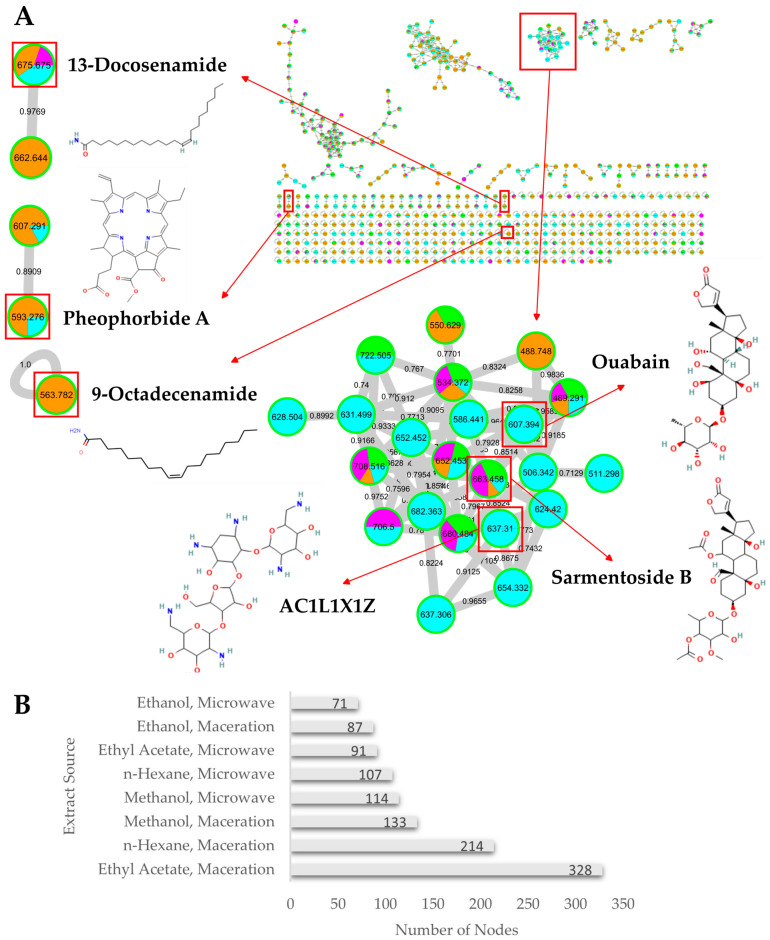
Profile putative compound of *S. duplicatum*’s extract. (**A**) The putative metabolite network of *S. duplicatum*’s extract, as generated by Cytoscape 3.10.3, comprising 567 nodes. Within this framework, 6 putative compounds with potential antimicrobial properties have been dereplicated, including 13-docosenamide,9-octadecanamide, pheophorbide A, ouabain, sarmentoside B, and AC1L1X1Z. Each color on the node represents the source of the extract: cyan (n-hexane), orange (ethyl acetate), green (methanol), and purple (ethanol), (**B**) The number of nodes based on the solvent and extraction method.

The bioactive compounds of *S. duplicatum* extracts from Kukup Beach were successfully characterized using LC-HRMS. The mass spectrum obtained showed similarities with the GNPS mass spectrum. The interactions formed were successfully visualized using Cytoscape 3.10.3. The visualization results showed the presence of a molecular network in each *S. duplicatum* extract. The interconnected nodes numbered 567 (21 in the positive node and 546 in the negative node) (Figure 1A), indicating a diverse array of compounds present in the samples, including fatty acids, terpenoids, alkaloids, and carbohydrates (Table 1). The largest number of nodes was found in *S. duplicatum* extracted with ethyl acetate through the maceration method, while the lowest number of nodes was found in *S. duplicatum* extracted with ethanol through the microwave method (Figure 1B).

The results of visualizing the putative compound network of *S. duplicatum* show variations in the number of nodes, which can range from a single node to multiple nodes, where a single node appears more frequently than the others (Figure 1A). For example, the putative compound 9-octadecanamide (*m/z* 563.782 [2M + H]) was represented by a single node. In contrast, two-node examples include the putative compounds 13-docosenamide (*m/z* 675.675 [2M + H]) and pheophorbide A (*m/z* 593.276 [M + H]). Additionally, multiple nodes were represented by compounds such as ouabain (*m/z* 607.394 [M + Na]^+^), sarmentoside B (*m/z* 663.458 [M + H]), and AC1L1X1Z (*m/z* 637.310 [M + Na]).

**Figure 2:**
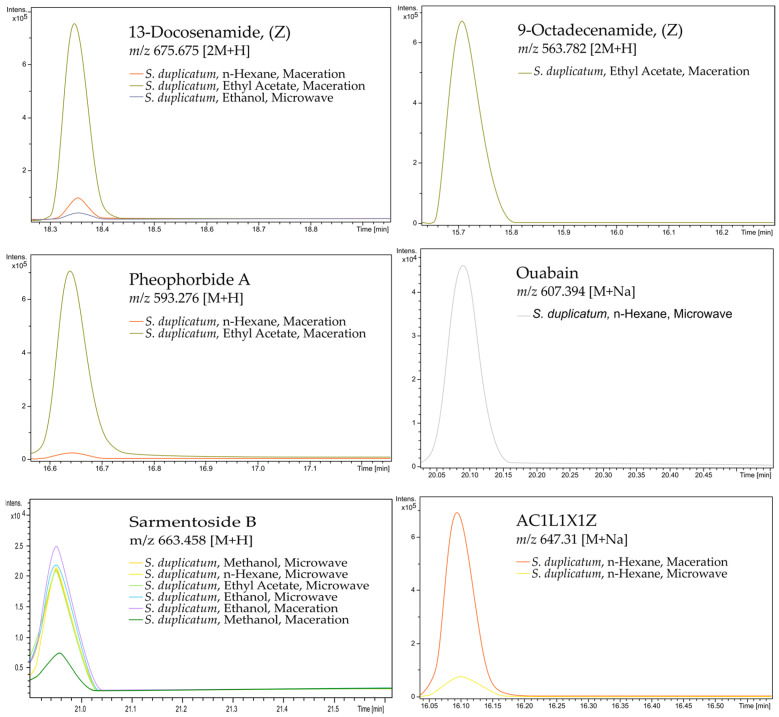
Retention time (RT), source, and intensity of putative compounds of *S. duplicatum*. RT, source, and intensity data were obtained from files (.d) processed in Compass Data Analysis 4.0 by extracting the parent mass chromatogram ions aligned with the GNPS database. The x-axis represents the RT of each compound, while the y-axis represents the compound intensity. Meanwhile, the color of each peak represents the source of the compound.

The retention time of putative compounds of *S. duplicatum* was successfully visualized through Compass Data Analysis 4.0 by extracting ion chromatograms from each parent mass (Figure 2). Each putative compound of *S. duplicatum* was identified at different retention times. The fastest retention time of the putative compound with potential as an antibacterial agent was found in the putative compound 9-octadecenamide, with a retention time of around 15.66–15.78 min. Meanwhile, the longest retention time of the compound with potential as an antibacterial was found in the putative compound sarmentoside B, with a retention time of around 20.92–21.02 min

**Figure 3:**
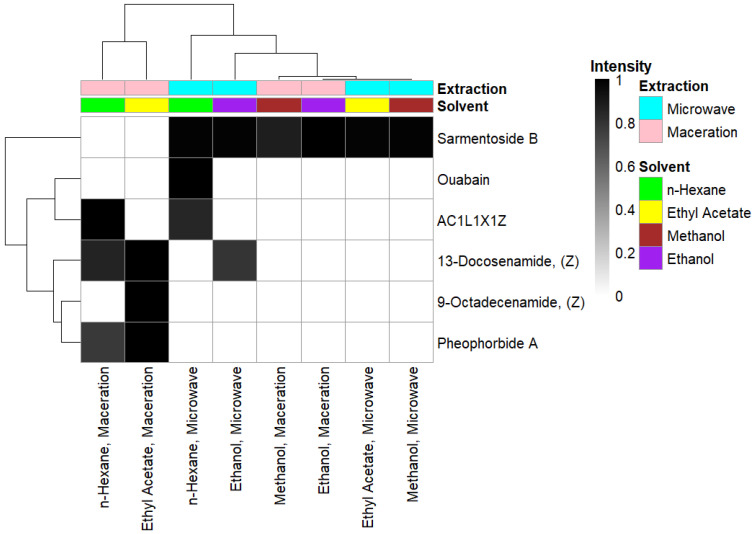
Hierarchical clustering analysis (HCA) and heatmap of putative compound intensities of *S. duplicatum*. All putative compound intensities were normalized to Min-Max. Ward’s method was used in the HCA formation, while bidirectional clustering was performed based on the putative compound intensities and the sources that could produce those putative compounds. In the heatmap, black and white colors indicate the highest and lowest intensities, respectively.

Meanwhile, the area under the peak at the retention time range indicates the relative intensity. The relative intensity value indicates the number of ions from the putative compound detected by the mass spectrometer. The relative intensity value of each putative compound of *S. duplicatum* is shown in Figure 3.

### 3.2. Antibacterial Bioassays

The antibacterial efficacy of *S. duplicatum* extract was evaluated against *S. aureus* ATCC 33592 (MRSA) and *E. coli* ATCC 35218 (a TEM-1 positive strain) using microbroth dilution assays. The test was conducted at four different extract concentration levels: 20.0, 10.0, and 5.0 μg/mL, with three repetitions per concentration. A summary of the results throughout the entire experimental data is presented in Table 2.

Among all the tested extracts, the ethyl acetate-maceration extract showed the strongest inhibitory activity, reaching inhibition values between 85 and 98% against *S. aureus*, while all other extracts were inactive against *S. aureus* ATCC 33592 and *E. coli* ATCC 35218 (Table 2). This significant difference (asymptotic significance < 0.05) was due to differences in solvents, with ethyl acetate having a more significant effect, with a mean rank of 16.92. Meanwhile, differences in the extraction method and concentration did not significantly affect the growth inhibition of *S. aureus* ATCC 33592, with asymptotic significance values of 0.583 and 0.309, respectively.

## 4. Discussion

### 4.1. LC-HRMS Metabolite Profiling and Molecular Networking

Among the identified putative metabolites of *S. duplicatum*, 13-docosenamide, ouabain, and sarmentoside B have been reported to have growth-inhibitory potential against various Gram-positive bacteria, including *Bacillus subtilis, Staphylococcus epidermidis*, and *S. aureus*. However, these putative compounds are less effective against Gram-negative bacteria, such as *Salmonella typhimurium, Salmonella enteritidis*, and *E. coli* [27,28,29]. This is likely due to the more complex and impermeable structure of the Gram-negative cell envelope, which may minimize their antibacterial effectiveness [30,31,32].

In contrast, the presence of the putative compound 9-octadecanamide showed an inhibitory effect against several Gram-negative pathogens, such as *Klebsiella pneumoniae* and *E. coli* [33,34]. The putative compound AC1L1X1Z was also reported to inhibit several Gram-negative bacteria, such as *Pseudomonas vulgaris, Pseudomonas aeruginosa*, and *E. coli*. In addition, AC1L1X1Z was also reported to inhibit several Gram-positive bacteria, such as *Enterococcus faecalis* and *S. aureus* [35]. This inhibition of Gram-positive and Gram-negative bacteria can also be carried out by the presence of the putative compound pheophorbide A, where the presence of the putative compound pheophorbide A can inhibit the growth of *S. aureus* and *E. coli* [36].

Interestingly, putative compounds in *S. duplicatum* with antibacterial potential can only be extracted using certain solvents and methods. For example, the putative compound 9-octadecanamide was only identified when *S. duplicatum* was extracted with ethyl acetate via maceration, while ouabain could only be extracted with n-hexane via microwave extraction. Microwave extraction also yielded the putative compounds sarmentoside B and AC1L1X1Z. Meanwhile, pheophorbide A were only extracted through maceration with n-hexane and ethyl acetate. The extract sources for each putative compound can be seen in Figure 2.

However, putative compounds with potential as antimicrobial agents are not only found in *S. duplicatum*, but also in other species, including 13-docosenamide, pheophorbide A, 9-octadecanamide, ouabain have been reported to be identified in *Sargassum polycystum, Sargassum cristaefolium*, *Sargassum granuliferum,* and *Macrocystis pyrifera*, respectively [37,38,39,40]. Meanwhile, sarmentoside B was first reported to be identified in *Strophanthus sarmentosus* [41], subsequently reported in the associated bacteria of *Stichopus vastus*, namely *Streptomyces cavourensis* [29]. The compound AC1L1X1Z was also reported in the phylum Actinobacteria, such as *Streptomyces fradiae* [42]. These two putative compounds have never been reported in seaweed. However, members of the phylum Actinobacteria have been reported as symbionts of *Sargassum* spp. [43,44,45], so these two compounds are likely derived from symbionts of *S. duplicatum.* Therefore, further isolation and identification of the putative compounds are needed for confirmation.

Each putative compound of *S. duplicatum* was identified at different retention times. The fastest retention time of the putative compound with potential as an antibacterial agent was found in the putative compound 9-octadecenamide. Meanwhile, the longest retention time of the compound with potential as an antibacterial was found in the putative compound sarmentoside B. The long retention time of sarmentoside B indicates that the putative compound sarmentoside B is difficult to elute when passing through the chromatogram column. These compounds can be extracted with one or more solvents via maceration or microwave. The intensity of each putative compound in *S. duplicatum* varies. Interestingly, the highest intensity is always achieved through maceration, as seen in the case of sarmentoside B and AC1L1X1Z. Furthermore, different solvents also affect the intensity of each suspected compound, as seen in the case of 13-docosenamide and pheophorbide A, where the highest intensity was achieved using ethyl acetate.

The variation in intensity can be caused by the polarity of the solvent used in the extraction process. For example, 13-docosenamide and pheophorbide A show higher intensities in the ethyl acetate extract. Furthermore, the extraction technique can also affect the recovery of putative compounds. In particular, the maceration method consistently produces the highest intensities for several putative compounds, such as sarmentoside B and AC1L1X1Z. This indicates increased efficacy for these specific analytes through longer solid–liquid contact at room temperature. Conversely, the lowest intensities of several putative compounds obtained through microwave extraction are likely due to the consequences of thermal degradation, where superheating can induce hydrolysis or structural denaturation of putative metabolites, particularly thermolabile putative compounds [46,47].

Furthermore, HCA also provided clear evidence that the chemical profile of *S. duplicatum* was significantly influenced by the extraction solvent and extraction method. The bidirectional clustering pattern showed that samples using the same solvent polarity tended to cluster together. This suggests that solvent type may be a major determinant in the recovery of putative metabolites. Notably, maceration and microwave extractions did not completely overlap in their clustering, suggesting that the extraction technique also contributed to differences in compound intensities. The differences in intensities of each extract were likely due to variations in energy input and cell disruption efficiency during the extraction process, particularly the microwave extraction method.

### 4.2. Antibacterial Bioassays

The high antibacterial activity of *S. duplicatum* extracted with ethyl acetate solvent through the maceration method is in line with the number and abundance of putative compounds, such as 13-docosenamide, 9-octadecenamide, pheophorbide A, and other putative compounds that have not been identified in the GNPS database (Figure 1 and Figure 3). Combinations of potential antibacterial compounds have been reported to produce a synergistic mechanism of action in inhibiting bacterial growth [48,49]. This mechanism may involve interference with bacterial DNA/RNA replication, such as with topoisomerase inhibitors, DNA ligase inhibitors, and DNA polymerase III inhibitors, thus inhibiting bacterial growth [50]. Bacterial growth inhibition can also occur through the inhibition of cell wall biosynthesis, such as the synthesis of the tessera and sacculus [51]. In addition, bacterial growth can also be inhibited by blocking protein synthesis and disrupting the bacterial cytoplasmic membrane [52,53].

## 5. Conclusions

The integration of antimicrobial assays with untargeted LC-HRMS metabolomics of *S. duplicatum* enabled efficient prioritization of active extracts and metabolites, reducing redundancy and focusing downstream isolation efforts on chemically unique and biologically relevant candidates. Metabolite profiling of *S. duplicatum* using LC-HRMS revealed the presence of several putative compounds with antimicrobial potential, such as 13-docosenamide, 9-octadecanamide, pheophorbide A, ouabain, sarmentoside B, and AC1L1X1Z. Furthermore, several putative compounds from *S. duplicatum* have not been identified in the GNPS database, indicating their potential novelty. Therefore, the isolation of these putative compounds is warranted in the future. Future studies should focus on the targeted isolation and purification of candidate metabolites identified through untargeted LC-HRMS, as well as mechanistic studies of antimicrobial action, to clarify whether these putative compounds act through novel modes of action. Furthermore, combining metabolomics with genomics, transcriptomics, and molecular networking will enhance compound annotation accuracy and facilitate biosynthetic pathway prediction.

## Figures and Tables

**Table 1:** Putative compounds identified from *S. duplicatum* exhibit potential as antimicrobial agents.

Ret. Time (min)	Compound Name	Compound Classification	Chemical Formula	Parent Mass	Adduct	Mol. Weight
18.30–18.40	13-Docosenamide	Fatty acids	C_22_H_43_NO	675.675	[2M + H]	337.6 g/mol
15.66–15.78	9-Octadecenamide	Fatty acids	C_18_H_35_NO	563.782	[2M + H]	281.5 g/mol
16.60–16.70	Pheophorbide A	Alkaloids	C_35_H_36_N_4_O_5_	593.276	[M + H]	592.7 g/mol
20.05–20.15	Ouabain	Terpenoids	C_29_H_44_O_12_	607.394	[M + Na]^+^	584.7 g/mol
20.92–21.02	Sarmentoside B	Terpenoids	C_34_H_48_O_13_	663.458	[M + H]^+^	664.7 g/mol
16.05–16.15	AC1L1X1Z	Carbohydrates	C_23_H_46_N_6_O_13_	637.310	[M + Na]	614.6 g/mol

**Table 2:** Antibacterial activity of *S. duplicatum* extracts by solvent and extraction method.

Extraction Method	Solvent	Concentration (μg/mL)	Inhibition (%)	
			*S. aureus* ATCC 33592	*E. coli* ATCC 35218
Maceration	n-Hexane	20.0	55	−2
		10.0	25	−1
		5.0	13	−1
	Ethyl acetate	20.0	85	−2
		10.0	98	−3
		5.0	96	−1
	Methanol	20.0	14	4
		10.0	11	4
		5.0	8	0
	Ethanol	20.0	−28	−2
		10.0	4	−5
		5.0	−23	−11
Microwave	n-Hexane	20.0	19	0
		10.0	18	−1
		5.0	12	−2
	Ethyl acetate	20.0	11	−2
		10.0	14	−1
		5.0	10	−1
	Methanol	20.0	11	−3
		10.0	28	−3
		5.0	−7	−3
	Ethanol	20.0	15	−3
		10.0	3	0
		5.0	1	−1

## Data Availability

The original contributions presented in the study are included in the article; further inquiries can be directed to the corresponding authors.
